# Supplementary material for: Whole exome sequencing study identifies candidate loss of function variants and locus heterogeneity in familial cholesteatoma
Source: PLoS One. 2023 Mar 15;18(3):e0272174. doi: 10.1371/journal.pone.0272174 (PMC10016674; doi:10.1371/journal.pone.0272174)
Supplement: S1 File — (PDF) [file pone.0272174.s001.pdf]

S1 Supporting Information. Supplementary Methods

For pipeline software versioning and associated data files see Table S1/S2. Default parameters were used unless otherwise stated. Reads were trimmed using TrimGalore and mapped using cgpMAP to GRCh38 to produce BAM files. Quality control (QC) steps were performed using the Picard toolkit (1) for marking of PCR duplicates (default options for removal of both optical and PCR duplicates) and hybridisation statistics. Variant Calling pipelines were based on the GATK germline variant caller (Haplotypecaller) and the other on Freebayes. The GATK pipeline follows the GATK best practice for germline short variant discovery for single samples workflow (https://gatk.broadinstitute.org/hc/en-us/articles/360035535932-Germline-short-variant-discovery-SNPs-Indels-). Both GATK-Haplotypecaller (2) and Freebayes (3) vcfs were filtered using a hard filtering strategy, which consist of choosing specific threshold for one or more annotation and removing those which do not meet thresholds. GATK variants were hard filtered using the parameters described here (https://gatk.broadinstitute.org/hc/en-us/articles/360035531112--How-to-Filter-variants-either-with-VQSR-or-by-hard-filtering). Freebayes variants were filtered using the bcftools filter function with the following criteria; depth (DP) >5, Number of alternate observations on the forward strand (SAF) / revere strand (SAR) > 0, Number of Reads Placed Right (RPR) or left (RPL) of > 1. Filtered variants from Freebayes and GATK were overlapped to give a consensus of high confidence variants for analysis. SNPs were overlapped using bcftools isec and indels using bedtools (>=10% overlap required), keeping VCF annotations from the Freebayes processed files for downstream analyses. VCF files were annotated with TOPMED and gnomAD GRCh38 files (as described https://github.com/brentp/slivar) (4). The following slivar impactful filtering strategy was used using the info and pass-only filters described here https://github.com/brentp/slivar/wiki/rare-disease. VCF files were also annotated using Ensembl-VEP (5) utilising the VEP GRCh38 cache supplemented with a 7-way PhastCons conservation bigwig file (hg38.phastCons7way.bw) (6). VCFs were also filtered for variants not within coding regions, SNPs with a conservation score < 0.1 and SIFT/PolyPhen not containing 'benign' or 'tolerated' predictions.

References

1. Institute B. Picard toolkit. Broad Institute, GitHub repository. 2018.
2. Poplin R, Ruano-Rubio V, DePristo MA, Fennell TJ, Carneiro MO, Van der Auwera GA, et al. Scaling accurate genetic variant discovery to tens of thousands of samples. BioRxiv. 2017:201178.

3. Garrison E, Marth G. Haplotype-based variant detection from short-read sequencing. arXiv preprint arXiv:12073907. 2012.
4. Pedersen BS, Brown JM, Dashnow H, Wallace AD, Velinder M, Tristani-Firouzi M, et al. Effective variant filtering and expected candidate variant yield in studies of rare human disease. NPJ Genomic Medicine. 2021;6(1):1-8.
5. McLaren W, Gil L, Hunt SE, Riat HS, Ritchie GRS, Thormann A, et al. The Ensembl Variant Effect Predictor. Genome Biology. 2016;17(1):122.
6. Pollard KS, Hubisz MJ, Rosenbloom KR, Siepel A. Detection of nonneutral substitution rates on mammalian phylogenies. Genome research. 2010;20(1):110-21.
7. Imai R, Sato T, Iwamoto Y, Hanada Y, Terao M, Ohta Y, et al. Osteoclasts Modulate Bone Erosion in Cholesteatoma via RANKL Signaling. J Assoc Res Otolaryngol. 2019;20(5):449-59.
8. Jovanovic I, Zivkovic M, Djuric T, Stojkovic L, Jesic S, Stankovic A. Perimatrix of middle ear cholesteatoma: A granulation tissue with a specific transcriptomic signature. The Laryngoscope. 2020;130(4):E220-E7.
