# Supplementary material for: Whole exome sequencing study identifies candidate loss of function variants and locus heterogeneity in familial cholesteatoma
Source: PLoS One. 2023 Mar 15;18(3):e0272174. doi: 10.1371/journal.pone.0272174 (PMC10016674; doi:10.1371/journal.pone.0272174)
Supplement: S1 Table — The number of reads mapped to the hg38 assembly was calculated to give aligned and unaligned statistics. Exome target coverage was calculated using the manufacturer’s bed files for DNA-seq library preps (see Material and methods). Maximum and mean coverage was calculated at target regions. The proportion of target regions with no coverage was also calculated. (DOCX) [file pone.0272174.s002.docx]

**S1 Table.** Alignment statistics for DNA-seq exome samples. The number of reads mapped to the hg38 assembly was calculated to give aligned and unaligned statistics. Exome target coverage was calculated using manufacturers bed files for DNA-seq library preps (see Material and methods). Maximum and mean coverage was calculated at target regions. The proportion of target regions with no coverage was also calculated.

| **Participant ID** | **Aligned reads** | **Unaligned reads** | **Total reads** | **Max target coverage** | **Mean target coverage** | **% Zero coverage targets** |
| --- | --- | --- | --- | --- | --- | --- |
| 1a | 64518480 | 127260 | 64645740 | 286 | 72.8 | 2.50% |
| 1b | 185667373 | 270975 | 185938348 | 386 | 141.3 | 2.30% |
| 2a | 82199584 | 266478 | 82466062 | 149 | 24.3 | 3.30% |
| 2b | 95789167 | 292509 | 96081676 | 184 | 27.2 | 3.30% |
| 3a | 67577637 | 116489 | 67694126 | 296 | 75.7 | 2.50% |
| 3b | 98061566 | 178742 | 98240308 | 296 | 99.1 | 2.50% |
| 3c | 41289262 | 72326 | 41361588 | 332 | 51.3 | 2.70% |
| 4a | 69108351 | 122949 | 69231300 | 293 | 75.5 | 2.40% |
| 4b | 131132020 | 214770 | 131346790 | 319 | 118 | 2.40% |
| 5a | 71198771 | 117503 | 71316274 | 289 | 78.5 | 2.50% |
| 5b | 24538405 | 46405 | 24584810 | 339 | 32.8 | 2.90% |
| 6a | 35228827 | 61703 | 35290530 | 272 | 43.4 | 2.80% |
| 6b | 42507685 | 81019 | 42588704 | 278 | 50.7 | 2.70% |
| 7a | 90036931 | 140691 | 90177622 | 555 | 108 | 2.50% |
| 7b | 84748883 | 157415 | 84906298 | 299 | 88.7 | 2.40% |
| 8a | 75046383 | 130609 | 75176992 | 531 | 96.9 | 2.60% |
| 8b | 57151816 | 64108 | 57215924 | 293 | 68.9 | 2.40% |
| 9a | 49073824 | 57200 | 49131024 | 282 | 62 | 2.60% |
| 9b | 73717554 | 143254 | 73860808 | 295 | 81.8 | 2.50% |
| 10a | 50820495 | 108847 | 50929342 | 286 | 58.7 | 2.70% |
| 10b | 87520498 | 160350 | 87680848 | 427 | 96.7 | 2.50% |
