# Supplementary material for: Whole exome sequencing study identifies candidate loss of function variants and locus heterogeneity in familial cholesteatoma
Source: PLoS One. 2023 Mar 15;18(3):e0272174. doi: 10.1371/journal.pone.0272174 (PMC10016674; doi:10.1371/journal.pone.0272174)
Supplement: S2 Table — (DOCX) [file pone.0272174.s003.docx]

**S2 Table. Bioinformatics tools and versions used to process variants.**

| **Resource** | **Source** | **Reference** |
| --- | --- | --- |
| cgpMAP (v3.2.0) | Cancer, Aging and Somatic Mutation group, Wellcome Sanger Institute | https://dockstore.org/containers/quay.io/wtsicgp/dockstore-cgpmap |
| Picard (v2.25.4) | Broad institute | http://broadinstitute.github.io/picard/¬† |
| GATK (v4.1.2.0) | - | Van der Auwera et al (2013) |
| VerifyBAMID (v1.1.3) | - | Jun et al (2012) |
| Bedtools (v2.30.0) | - | Quinlan et at (2010) |
| Samtools (v1.12) | - | Danecek et al (2021) |
| Slivar (v0.2.2) | - | Perderson et al (2021) |
| Ensembl VEP (v104) | - | McClaren et al (2016) |
| TRAPD | - | Guo et al (2018) |
| Freebayes (v1.3.5) | - | Garrison et al (2012) |
