# Supplementary material for: Whole exome sequencing study identifies candidate loss of function variants and locus heterogeneity in familial cholesteatoma
Source: PLoS One. 2023 Mar 15;18(3):e0272174. doi: 10.1371/journal.pone.0272174 (PMC10016674; doi:10.1371/journal.pone.0272174)
Supplement: S3 Table — (DOCX) [file pone.0272174.s004.docx]

**S3 Table**. **A list of the files and their versions used by the bioinformatics tools.**

| **Software** | **Data files** | **Version** |
| --- | --- | --- |
| VEP | GENCODE | GENCODE 38 |
| - | PolyPhen | v2.2.2 |
| - | genebuild | 2014-07 |
| - | SIFT | sift5.2.2 |
| - | Conservation | PhastCons7way |
| Slivar | gnomAD | hg38 (v2) |
| - | TOPmed | hg38 dbSNP 151 |
| GATK | known-sites | hg38 1000G phase1 snps high confidence |
| - | known-sites | hg38 Mills and 1000G gold standard indels |
| - | known-sites | hg38 dbSNP 138 |
| TRAPD | gnomAD | r2.0.2 |
