# Supplementary material for: Whole exome sequencing study identifies candidate loss of function variants and locus heterogeneity in familial cholesteatoma
Source: PLoS One. 2023 Mar 15;18(3):e0272174. doi: 10.1371/journal.pone.0272174 (PMC10016674; doi:10.1371/journal.pone.0272174)
Supplement: S4 Table — Genes identified from the family overlap and mutation burden analysis (TRAPD) were overlapped with genes that were significantly under-expressed in the transcriptomics studies from Imai et al (2019) or Jovanovic et al (2020). (DOCX) [file pone.0272174.s005.docx]

**S4 Table. Underexpressed and mutated genes.** Genes identified from the family overlap and mutation burden analysis (TRAPD) were overlapped with genes that were significantly under-expressed in the transcriptomics studies from Imai *et al* (2019)(7) or Jovanovic *et al* (2020)(8).

| **rsID** | **gnomAD popmax AF** | **TOPMED AF** | **Gene** | **Consequence** | **SIFT** | **PolyPhen** | **Conservation** | **Families** |
| --- | --- | --- | --- | --- | --- | --- | --- | --- |
| rs199958643 | 0.001 | 7.17E-05 | *PPL* | missense | deleterious | probably damaging | 0.994 | S01 |
| rs755131245 | 0.000 | 4.78E-05 | *TJP3* | missense | deleterious | possibly damaging | 0.991 | S08 |
| rs144220426 | 0.001 | 3.98E-04 | *COL17A1* | missense | deleterious low confidence | probably damaging | 1.000 | S10 |
| rs35873579 | 0.007 | 1.98E-03 | *CYP24A1* | missense | deleterious | probably damaging | 0.928 | S12 |
| rs201453310 | 0.008 | 5.50E-03 | *MUC16* | missense | deleterious low confidence | probably damaging | 0.362 | S53 |
| rs147267769 | 0.004 | 2.57E-03 | *MMP10* | stop gained | NA | NA | 0.986 | S05 |
