# Supplementary material for: Whole exome sequencing study identifies candidate loss of function variants and locus heterogeneity in familial cholesteatoma
Source: PLoS One. 2023 Mar 15;18(3):e0272174. doi: 10.1371/journal.pone.0272174 (PMC10016674; doi:10.1371/journal.pone.0272174)
Supplement: Attachment — Submitted filename: Response to Plos One Peer Review December 2022.docx [file pone.0272174.s010.docx]

# Response to Reviewers by the Authors:

Thank you for the careful review and suggestions for producing a revised final manuscript.

We have incorporated changes into the manuscript in response to each of the reviewer comments below, which has improved clarity and detail.

The revised version of the manuscript also has edits that were made while proof-reading the manuscript, and with reference to the journal’s style requirements.

**Reviewer #1:**

Recommend to be cautious when concluding anything from this very explorative study.

Recommend in line 403 and 403 to find an alternative and less strong word than 'Evidence'

- **Author response: we agree that a circumspect approach is needed to interpret these early findings from whole exome analysis and have edited that sentence that you suggest in the discussion to be more tentative.**

***“The agreement between cholesteatoma functional profiling and gene expression data suggests that the deleterious variants described are likely to have contributed to the disease.”***

The authors provide analyses within families, between families and between/across single participants. Which of these is the main priority of the study? Explain your motivation for- and discuss the implications of all three approaches (interpretation of the results).

- **Author response: the study was designed to allow (1) overlap analysis within families to identify candidate (rare, loss of function) variants that co-segregate with disease (2) subsequent overlap analysis between families to identify any generalisable findings about common genes and pathways associated with cholesteatoma. The family-level overlap analysis is important for filtering out as many variants that are not linked to cholesteatoma as possible – given the large number of rare variants that are picked up in any WES analysis for an individual. We share the full list of 398 rare LOF variants identified in the family level study in supplementary table S1. We also expect that the primary WES data from individual participants, coupled with their family history data, will be of interest to other researchers for additional analyses – therefore we present the accession numbers in table 1 so that our data is available to the science community via the EGA repository.**
- **Thank you for this query - because of it, we have edited the first paragraph of the discussion (see below) to be clearer and congruent with our study design section of the Materials and Methods.**

***“******The primary aim of this study was to identify candidate genetic variants that co-segregate with cholesteatoma within and between families. Bioinformatic analysis was used to annotate the genes of interest, which may have a role in cholesteatoma pathology.”***

Cholesteatoma mostly occurs sporadically - not inheritably. Everything that leads to chronic ear disease (e.g. mucosal or skin diseases) can indirectly increase the risk for cholesteatoma. This is a point that I recommend to add when discussing rare familial/inherited genetic variants with consequences for e.g. cilial function - and the link between this and cholesteatoma.

- **Author response: We agree with the reviewer that, as a complex trait, cholesteatoma is likely to have a multifactorial etiology and that most cases present sporadically with no previous FH. Genetic studies like this one (that focus on rare families with extreme phenotypes and with multiple affected members) are of use for identifying the broad causal biological pathways that contribute to pathology. And we do not expect any candidate genetic change will be essential or sufficient to cause the disease. For conditions with heterogeneous underlying biological pathways and endophenotypes – constitutional (genetic) factors; environmental factors; and random events will all have a role. Studies like this one can only begin to unpick any genetic architecture involved in the pathology but may also identify broad areas of biology that can also be affected by environmental factors.**
- **To acknowledge this issue, we have edited the suggested section about cilial function which now reads:**

***“******These findings suggest that genetic factors that alter cilia structure and function may contribute to the development of some cases of cholesteatoma. Other non-constitutional risk factors and different disease pathways are inevitable given that most cases of cholesteatoma are sporadic cases and the complexity of the phenotype.”***

Emphasize, that this is an explorative study and discuss the false discovery rate/ likelihood of incidental findings in these small groups.

- **Author response: Again, we agree with the reviewer that a circumspect approach is appropriate in presenting these findings. We have added the following section to the start of the limitations section in our discussion,**

***“******There are some limitations to discuss, we describe a hypothesis-generating observational study of exome data from 21 participants, and so there is a risk of both false discovery (type 1 error) and of missing variants of interest (type 2 error).”***

- **We think this new phrase complements and introduces the elaborations of limitations already described in that section of the manuscript.**

**Reviewer #2:**

The authors addressed familial cholesteatoma genetic architecture via whole exome sequencing (WES) for 21 individuals treated for cholesteatoma recruited from ten affected families. They searched for single nucleotide variants, insertions and deletions and performed a hard filtering to these variants to obtain high confidence variants. They fetched for variants that are shared between affected individuals within families. They performed gene-level mutational burden analysis and enrichment analyses. They detected the genes that showed rare loss of function (LOF) variants and identified six genes (DENND2C, DNAH7, NBEAL1, NEB, PRRC2C, and SHC2) which have LOF variants in two or more families.

Functional enrichment analysis of the detected genes revealed 6 common pathways including calcium ion binding, extracellular matrix (ECM) organization, and GTPase activity. The missense variants were analysed to predict the impact on protein functionality. All variants were classified as damaging/deleterious missense variants. One of DNAH7 variants (rs115474479) is classified as an indel (stop gained) mutation. Authors suggest that DNAH7 variants are of interest because they encode a protein component of human cilia, whose functional mutations have been associated with primary ciliary dyskinesia (PCD) which accompanies Cholesteatoma. The study used multiple bioinformatics tools in for analysis.

There are some concerns for the improvement of the manuscript.

1. Please, mention the number of participants and number of corresponding families in the materials and methods section.

- **Author response: Thank you – edits were made to the sub-section Setting, Research Governance, and Participants.**

***“A family history was collected from the index case of 10 families and any relatives who subsequently joined the study. For each family member recruited, we recorded on a REDCap (22) database the following: relationship to index case; date of birth; age at diagnosis and/or age at time of surgery; unilateral or bilateral disease; secondary otology phenotypes; and diagnosis of genetic disease/congenital disorders.***

***Biological samples and DNA extraction***

***Blood samples from 21 participants were collected in 3ml EDTA tubes and DNA extracted using the QIAamp DNA Blood Mini Kit (Qiagen, UK).”***

2. Would you please, mention the storage conditions of samples (line 160)?

- **Author response: Thank you – edits were made to the sub-section Biological Samples and DNA extraction.**

***“Before DNA extraction and quantitation was completed, samples were stored at 4 °C. Purified DNA was stored at - 80 °C.”***

3. Please, include fig S1 in the main figures as it shows the 6 common pathways of genes obtained by the two methods of analyses. Please, add a figure caption for it and unify the way of writing the labels (Upper case or lower case for the first letter).

- **Author response: Thank you – edits were made to the sub-section of the Results – Mutational Burden Analysis. Please see the new Figure 4, with a detailed legend in the main manuscript (replacing Figure S1).**

***“Fig 4. Common pathways enriched. Common pathway and ontology terms found to be enriched for genes containing deleterious variants (p < 0.01; Hypergeometric test) in both the family overlap (red) and TRAPD (blue) analysis. The number of genes with deleterious variants in each pathway or ontology term are shown.”***

4. Lines 321-323: ‘Calcium ion binding’ is repeated in the following part ‘’ These 6 included calcium ion binding, extracellular matrix (ECM) organization, GTPase activity and calcium ion binding, containing a larger number of genes for each term in the mutational burden analysis (Fig S1)'', so please correct it.

- **Author response: Thank you – paragraph was corrected.**

***“These six included extra-cellular matrix (ECM) organization, GTPase activity, and calcium ion binding; each containing a larger number of associated genes in the mutational burden analysis compared to the family overlap analysis (Fig 4).”***

5. There are some typos and punctuation errors need to be corrected.

- **Author response: Thank you – proof reading and edits were made throughout the manuscript (including edits to sub-headings and technical nomenclature) in addition to the changes described above, which are highlighted in red in our annotated revised manuscript.**
