# Supplementary material for: Whole exome sequencing study identifies candidate loss of function variants and locus heterogeneity in familial cholesteatoma
Source: PLoS One. 2023 Mar 15;18(3):e0272174. doi: 10.1371/journal.pone.0272174 (PMC10016674; doi:10.1371/journal.pone.0272174)
Supplement: Attachment — Submitted filename: Plos One Response Jan23.docx [file pone.0272174.s011.docx]

We thank the reviewers for their comments. We have responded to each point raised by the reviewers below and detailed changes we have made to the manuscript.

Response to Reviewer #1

No further comments.

**Author response: We thank the reviewer for taking the time to read through our manuscript and we are happy that our previous changes are acceptable.**

Response to Reviewer #2

1) Regarding figure 4 “Fig 4. Common pathways enriched. Common pathway and ontology terms found to be enriched for genes containing deleterious variants (p < 0.01; Hypergeometric test) in both the family overlap (red) and TRAPD (blue) analysis. The number of genes with deleterious variants in each pathway or ontology term are shown.”

Is there a significant difference between the number of genes of family overlap (red) and TRAPD (blue) in each pathway? Can you show this on the bar chart and the error bars ?

**Author Response: We thank the reviewer for their comment and have added additional information to Figure 4. Figure 4 uses gene counts for each gene ontology term or pathway. Each GO/pathway therefore only contains one count value (i.e. 358 genes from the TRAPD analysis in the ion binding pathway), therefore there is no spread to be visualised using error bars. We have performed an additional analysis comparing the numbers in the two analysis and adding onto Figure 4 the p-value for those pathways where there is a significant higher number of genes in the TRAPD analysis compared to the “family overlap”. We have also added the following detail in the figure legend:**

*“Pathway and ontology terms where there is a significant increase in the genes associated with that pathway in the TRAPD analysis compared to the overlap analysis are highlighted (p<0.05; one-sided 2-sample test for equality of proportions with continuity correction).”*

2) According to your findings do you have any future perspectives concerning the study of genetic variants in cholesteatoma within and between families?

**Author Response: We think the study of genetic variants in cholesteatoma within and between families should be expanded and complemented, given that any genetic architecture can be expected to be complex and heterogeneous.**

**In the final two sentences of our discussion, we express the need for more studies to identify candidate variants (given that our work to date has not been exhaustive for the reasons outlined in the limitations section).**

**More work should include**

- **Family studies in large multiply affected pedigrees**

**These could also identify more variants of interest through the use of WGS in addition to WES, and should be designed to consider the penetrance of candidate variants, through sequencing affected and unaffected individuals. These types of study would build on our own linkage approach that includes the most distantly related individuals and used WES rather than more comprehensive WGA.**

- **Cohort and case control analysis of unrelated, affected individuals to identify candidate variants of interest.**

**As mentioned in the discussion, we are now using a GWAS approach with WES data collected by UK Biobank.**

**Thank you for this question, we have added an additional clarifying sentence to the end of our discussion in response.**

*Therefore, our search for candidate pathogenic variants cannot be considered exhaustive and should be expanded in studies of large, affected pedigrees to identify more variants of interest, and to consider the penetrance of candidate variants.*

*Our findings will now be applied to an analysis of sequencing data from a much larger cohort of individuals treated for cholesteatoma and recruited to UK Biobank (51).*

3) Please, revise the typos.

**Author response: We have corrected the typos identified and have found other typos by getting additional people to read the document and passing the document through two document checking algorithms (grammar.ly and LanguageTool). Hopefully, all issues have now been found.**
